# Contralesional Sensorimotor Network Participates in Motor Functional Compensation in Glioma Patients

**DOI:** 10.3389/fonc.2022.882313

**Published:** 2022-04-22

**Authors:** Shengyu Fang, Lianwang Li, Shimeng Weng, Yuhao Guo, Zhang Zhong, Xing Fan, Tao Jiang, Yinyan Wang

**Affiliations:** ^1^ Department of Neurosurgery, Beijing Tiantan Hospital, Capital Medical University, Beijing, China; ^2^ Beijing Neurosurgical Institute, Capital Medical University, Beijing, China; ^3^ Research Unit of Accurate Diagnosis, Treatment and Translational Medicine of Brain Tumors, Chinese Academy of Medical Sciences, Beijing, China

**Keywords:** resting-state functional magnetic resonance images, graph theory, brain reorganization, topological property, glioma

## Abstract

**Background:**

Some gliomas in sensorimotor areas induce motor deficits, while some do not. Cortical destruction and reorganization contribute to this phenomenon, but detailed reasons remain unclear. This study investigated the differences of the functional connectivity and topological properties in the contralesional sensorimotor network (cSMN) between patients with motor deficit and those with normal motor function.

**Methods:**

We retrospectively reviewed 65 patients (32 men) between 2017 and 2020. The patients were divided into four groups based on tumor laterality and preoperative motor status (deficit or non-deficit). Thirty-three healthy controls (18 men) were enrolled after matching for sex, age, and educational status. Graph theoretical measurement was applied to reveal alterations of the topological properties of the cSMN by analyzing resting-state functional MRI.

**Results:**

The results for patients with different hemispheric gliomas were similar. The clustering coefficient, local efficiency, transitivity, and vulnerability of the cSMN significantly increased in the non-deficit group and decreased in the deficit group compared to the healthy group (*p* < 0.05). Moreover, the nodes of the motor-related thalamus showed a significantly increased nodal efficiency and nodal local efficiency in the non-deficit group and decreased in the deficit group compared with the healthy group (*p* < 0.05).

**Conclusions:**

We posited the existence of two stages of alterations of the preoperative motor status. In the compensatory stage, the cSMN sacrificed stability to acquire high efficiency and to compensate for impaired motor function. With the glioma growing and the motor function being totally damaged, the cSMN returned to a stable state and maintained healthy hemispheric motor function, but with low efficiency.

## Highlights

This study investigated how the contralesional hemispheric sensorimotor network (cSMN) was altered in glioma patients with different motor status (deficit or normal). Two potential stages (compensated and decompensated) of motor function alteration were found. When the glioma initially appeared and the motor function of patients was normal, the cSMN sacrificed stability to acquire high efficiency in order to compensate for the impaired motor function induced by glioma. With the glioma growing and the degree of disruption of motor function being high, the damaged motor function was unable to be compensated by the cSMN; the cSMN, therefore, returned to a stable state and maintained healthy hemispheric motor function, but with low efficiency. Our findings verified that the contralesional hemispheric cortex participated in the motor functional compensation through altering the functional brain network.

## Introduction

Motor function is essential for daily life, and motor dysfunction induced by a glioma has a major impact on a patient’s quality of life (1). Owing to neural plasticity and functional compensation, some deficits can be recovered (2–4). Normal cortices surrounding lesions (5) and mirror-symmetrical cortices in the contralesional hemisphere are recruited to compensate for the dysfunction (4–6). However, since primary sensorimotor cortices are responsible for a sole function, remodeling through surrounding areas and then compensating for the damaged motor function are difficult (7). Accordingly, it becomes crucial to investigate network remodeling in the mirror-symmetrical sensorimotor cortices to understand the compensation for motor deficits.

Many multimodal imaging studies have demonstrated that cortical compensation in the contralesional hemisphere was related to functional recovery (8–11). Glioma is a progressive disease. It is probable for a glioma that grew on the sensorimotor network to induce preoperative motor deficit, but not all gliomas do. Cortical destruction and reorganization are related to this phenomenon, but the detailed mechanisms remain unclear. Hence, we investigated how alterations of the contralesional sensorimotor network (cSMN) in patients with different preoperative motor status help in understanding the mechanisms of motor functional compensation.

Resting-state functional MRI (rs-fMRI) is widely used to delineate functional networks. Accumulating rs-fMRI studies investigated the correlations between alterations of functional connectivity (FC) and glioma-induced functional plasticity in glioma patients (8, 12). In addition to FC, topological properties are another dimension to delineate network connections (13), which show the hierarchical connections of nodes in a network and represent the characteristics of information conveying (14, 15). Thus, the topological properties of functional networks are expected to provide a new perspective in explaining functional compensation. The current study aimed to 1) reveal how the cSMN is altered in patients with gliomas that have invaded sensorimotor-related structures and 2) identify relevant changes between the topological properties and preoperative motor deficits by comparing patients with different motor status. Subsequently, we hoped to provide some evidence for recovery treatments by identifying crucial nodes of the cSMN.

## Materials and Methods

This study was reviewed and approved by the institutional review board of the Beijing Tiantan Hospital. Written informed consent was obtained from all patients for their original treatments.

### Participants

We retrospectively reviewed the medical records of 70 patients who were diagnosed with gliomas in sensorimotor function-related areas between August 2017 and March 2020 at Beijing Tiantan Hospital. The inclusion criteria were as follows: 1) adult patients and 2) a primary glioma without any history of treatment. The exclusion criteria were as follows: 1) head motion >1 mm in translation or 1° in rotation, 2) lesions located bilaterally, and 3) tumor resulting in midline shifting.

All enrolled patients were first divided based on tumor location and were subsequently classified into a deficit and a non-deficit group based on their preoperative motor status. The muscle strength of each patient was tested using the British Medical Research Council scale and by neurosurgeons who have over 10 years of work experience in clinical neurosurgery. If the muscle strength of any limbs was lower than grade 5, the patient would be defined as having preoperative motor deficit. After matching for age, sex, and educational status, 33 healthy participants were enrolled as controls (men, *n* = 18). The handedness of all patients and participants was examined using the Edinburgh Handedness Inventory test. All of them were right-handed.

### MRI Acquisition

In the current study, a Siemens 3.0-T MR scanner (MAGNETOM Prisma, Erlangen, Germany) was used to collect imaging data. The parameters for the T1 magnetization-prepared rapid acquisition gradient echo with gadolinium enhancement were as follows: repetition time (TR) = 2,300 ms, echo time (TE) = 2.3 ms, field of view (FOV) = 240 × 240 mm^2^, flip angle (FA) = 8°, slice number = 192, and voxel size = 1.0 × 1.0 × 1.0 mm^3^. The parameters for the T2-weighted fluid-attenuated inversion recovery (T2-FLAIR) sequence were: TR = 5,000 ms, TE = 387 ms, FOV = 220 × 220 mm^2^, FA = 150°, slice number = 128, thickness = 0.9 mm, and voxel size = 0.4 × 0.4 × 0.9 mm^3^.

The rs-fMRI images were acquired with an echo planar imaging sequence. The parameters were as follows: TR = 2,000 ms, TE = 30 ms, FOV = 220 × 220 mm^2^, FA = 90°, slice number = 30, voxel size = 3.0 × 3.0 × 3.0 mm^3^, and acquisition duration = 8 min. Participants were asked to get monitored without thinking about anything during rs-fMRI acquisition.

### Regions of Tumor Invasion

**Figure 1:**
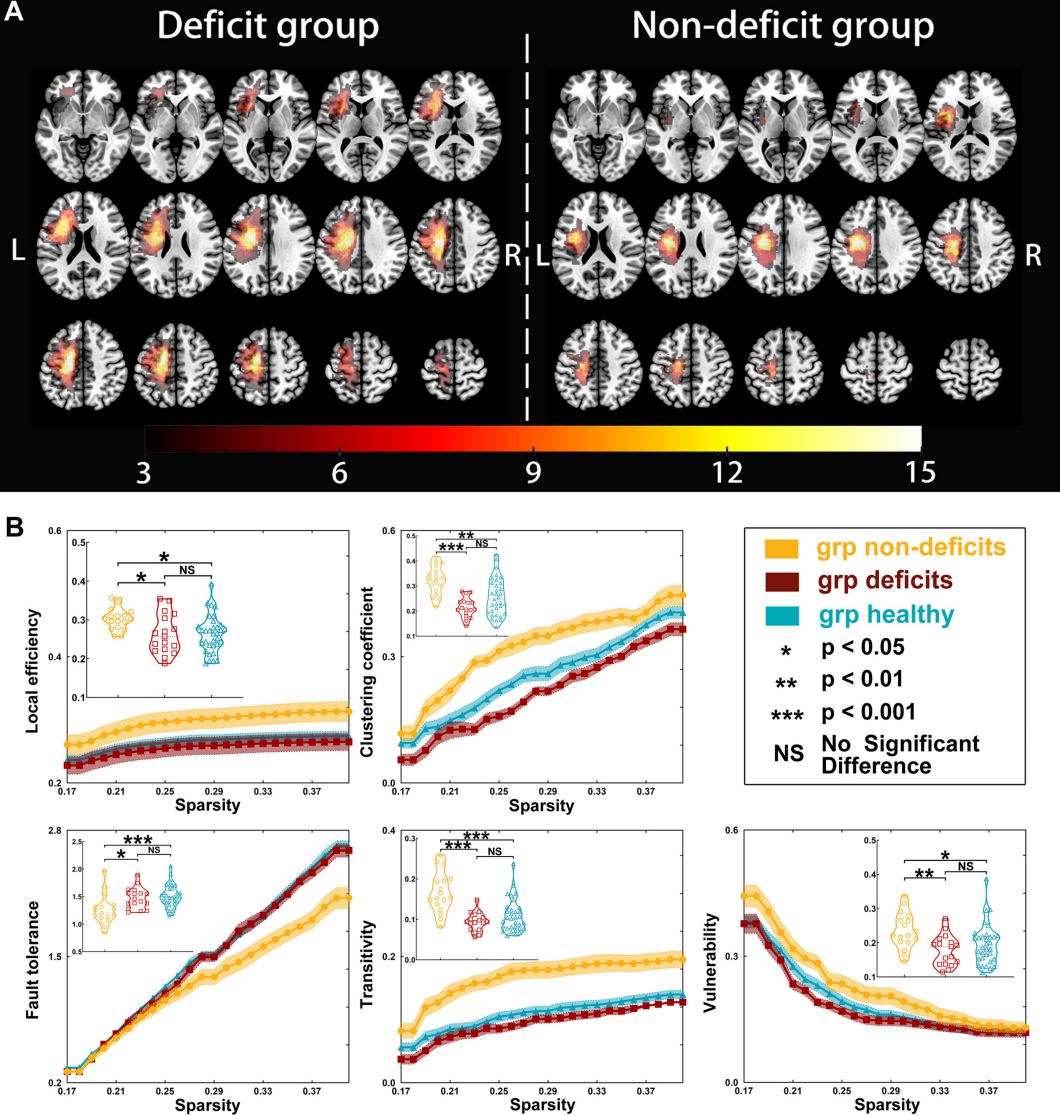
Tumor location and global properties of patients with left hemispheric gliomas. **(A)** Overlapping results of gliomas in the left hemisphere. The *value of the color bar* represents the number of patients with tumors located in a same region. **(B)** Differences in global topological properties of the sensorimotor network in the contralesional hemisphere among the three groups.

**Figure 2:**
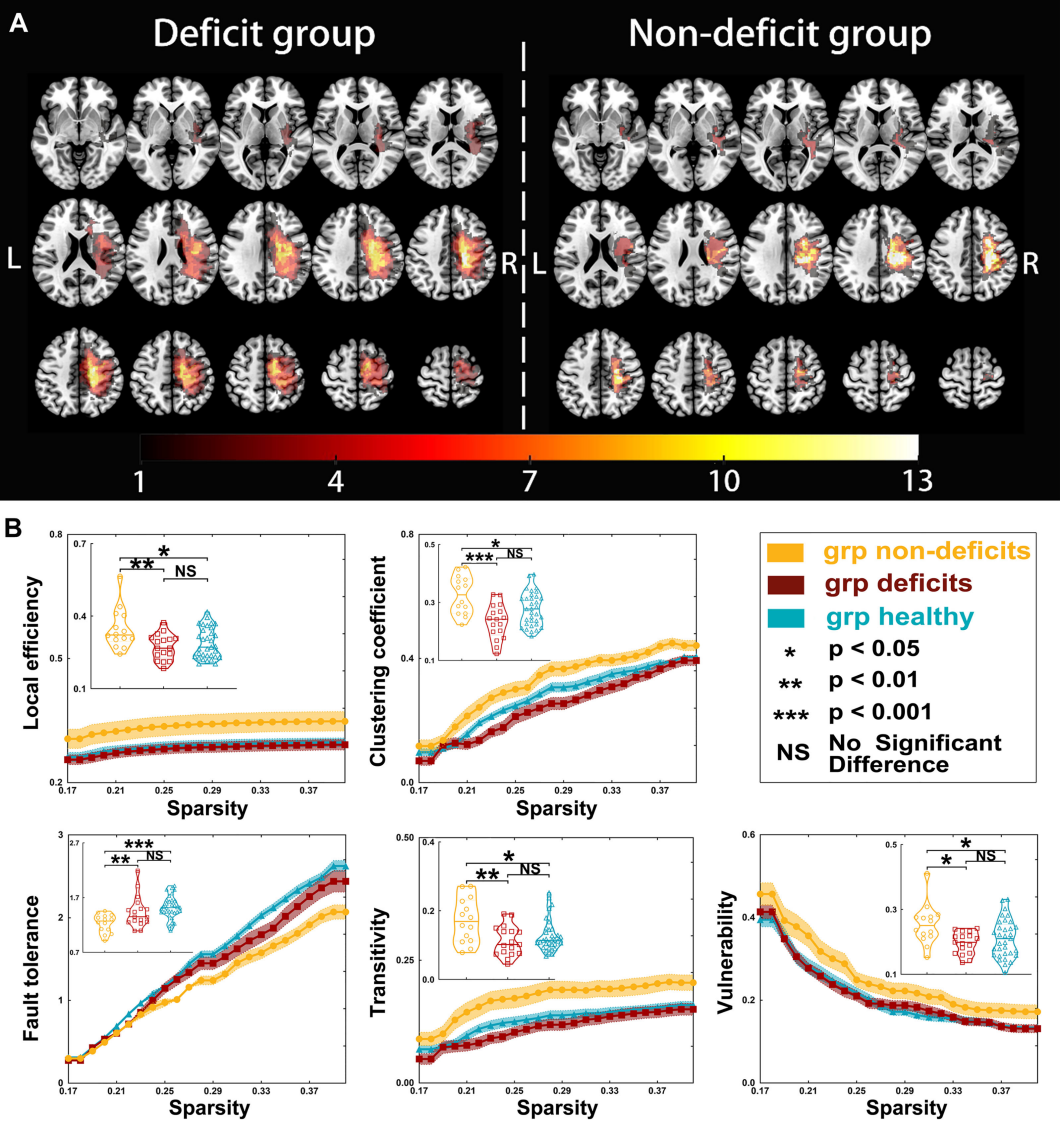
Tumor location and global properties of patients with right hemispheric gliomas. **(A)** Overlapping results of gliomas in the right hemisphere. The *value of the color bar* represents the number of patients with tumors located in a same region. **(B)** Differences in global topological properties of the sensorimotor network in the contralesional hemisphere among the three groups.

Two neuroradiologists independently reviewed the MR images using MRIcron software (http://www.mccauslandcenter.sc.edu/mricro/mricron/) to manually determine the extent of glioma invasion (shown in **Figures 1**, **2**) (16). The tumor masks of lower-grade glioma (grades II and III) and glioblastoma (grade IV) were drawn based on the high-intensity regions of FLAIR and the enhancement regions of the contrast-enhanced T1-weighted images, respectively. If there was a more than 5% difference in the region drawn by the two neuroradiologists, a third neuroradiologist with 25 years of experience made the final decision. All tumor masks were normalized to the MNI-152 T1 template using SPM 8 software (University College London, London, UK; http://www.fil.ion.ucl.ac.uk/spm/). The tumor volumes were calculated with a volumetric measurement.

### Functional MRI Preprocessing

We used GRETNA (https://www.nitrc.org/projects/gretna) (17, 18) to preprocess the rs-fMRI data. Information of the preprocessing pipeline and the parameters in each step were as follows: 1) transformation to a NIFTI file; 2) removal of the first images (time point number to remove = 5); 3) slice timing correction; 4) realignment; 5) spatial normalization (normalized to echo planar imaging template) (19); 6) smoothing (full width at half maximum = 4 mm); 7) temporal detrending (linear detrending); 8) regressing out the covariance (white matter signal: with WMMask_3mm; CSF signal: with CSFMask_3mm; head motion: Friston, 24 parameters); 9) temporal filtering (0.01–0.08 Hz); and 10) scrubbing (using default parameters and the interpolation strategy: linear interpolation; FD threshold, 0.5; previous time point number, 1; subsequent time point number, 2).

### Regions of Interest

Tumor occupation can induce a mismatch during the step of normalization, and neurovascular uncoupling decreases the accuracy of blood oxygen signals (20–22). Accordingly, the results related to the lesional sensorimotor network would be inaccurate with the effects of these factors. To avoid these effects, the regions of interest in the cSMN that were unrelated to these factors, to keep our results reliable, were extracted from a brain atlas (http://www.brainnetome.org/) (23) using the BRANT software. The seeds were generated as 5-mm spheres based on the coordinates of the sensorimotor network. Finally, 14 nodes were extracted to construct a cSMN template (**Supplementary Tables S1, S2**).

### Network Construction

Pearson’s correlation coefficients were applied to construct the FC matrices by calculating the regional mean time series for all extracted nodes of the sensorimotor networks.

### Graph Theoretical Measures

Graph theoretical measurement is a reliable method to quantitatively reveal topological properties (24, 25). In this study, all weighted matrices were transformed into absolute to calculate the topological properties, including local efficiency, clustering coefficient, global efficiency, the shortest path length, small-worldness (gamma, lambda, and sigma), fault tolerance, transitivity, vulnerability, nodal efficiency, nodal local efficiency, nodal cluster coefficient, and betweenness centrality (24, 26–28). Detailed information of each property is shown in the **Supplementary Material**.

### Statistical Analyses

We used SPSS 25.0 software (Microsoft^®^, IBM Corp., Armonk, NY, USA) to perform statistical analyses. The clinical characteristics were compared between the patient and control groups using Student’s *t*-test, Mann–Whitney *U* test, chi-square test, and one-way ANOVA according to the type of data. To explore group differences in network topological properties, we applied a series of sparsity thresholds (from 0.17 to 0.40, interval = 0.01) consistent with the literature (29). We selected the tumor volume and malignancy grade as covariates during the comparison of the FCs and topological properties among the groups. Bonferroni correction was used to correct the FC results. The topological properties were compared among the groups using one-way ANOVA. Bonferroni correction was subsequently used for *post-hoc* analysis when the results of one-way ANOVA were significantly different. A *p*-value <0.05 was considered as significant. Furthermore, we analyzed the correlation between the preoperative muscle strength of deficit hand and topological properties using Spearman’s correlation.

## Results

Sixty-five right-handed patients (men, *n* = 32) met the inclusion criteria. The preoperative Karnofsky performance scores of the non-deficit group were higher than those of the deficit group (*p* < 0.001). Moreover, the tumor volumes were larger in the deficit group than those in the non-deficit group (left hemisphere, *p* = 0.016; right hemisphere, *p* = 0.033). No differences were observed in age, sex, or educational status among the three groups (**Table 1**). The differences in FC are shown in the **Supplementary Material**.

**Table 1:** Demographic and clinical characteristics.

Demographic and clinical characteristics	Left hemisphere		Right hemisphere		Healthy (*n* = 33)	Left hemisphere	Right hemisphere
	Non-deficit group (*n* = 17)	Deficit group (*n* = 17)	Non-deficit group (*n* = 14)	Deficit group (*n* = 17)		*p*-value	*p*-value
Gender							
Male	8	10	6	8	18	0.782	0.735
Female	9	7	8	9	15		
Age (years)*	40.4 ± 2.2	39.8 ± 3.0	41.8 ± 3.4	43.4 ± 2.2	37.2 ± 1.5	0.487	0.088
Handedness							
Right	17	17	14	17	33	–	–
Left	0	0	0	0	0		
KPS score (preoperative)							
100	15	0	5	0	33		
90	2	0	9	0	0	<0.001	<0.001
80	0	14	0	14	0		
70	0	3	0	3	0		
Motor deficit duration (months)	–	1.9 ± 0.3	–	2.3 ± 0.4	–	–	–
Education period (years)*	13.5 ± 0.8	13.4 ± 0.7	12.5 ± 0.7	13.2 ± 0.82	13.4 ± 0.6	0.994	0.654
Tumor grade					–		
II	6	4	7	7		0.708	0.725
III	11	13	7	10			
Tumor volume (ml)*	57.66 ± 8.66	92.39 ± 10.54	60.63 ± 7.71	87.98 ± 9.08	–	0.016	0.033

Motor deficit duration was the time from outpatient diagnosis to inpatient functional MRI scan.

KPS, Karnofsky Performance Scale.

*Values are the mean ± SEM. Student’s t-test was used to compare the differences of the tumor volume and the Karnofsky Performance Scale scores between the deficit and non-deficit groups. One-way ANOVA was used to compare the differences of age and education period between the deficit, non-deficit, and healthy groups. Fisher’s test was used to compare the differences of gender and tumor grade between the deficit and non-deficit groups. The deficit group comprised patients with preoperative motor deficit; the non-deficit group was composed of patients without preoperative motor deficit.

### Differences in Global Topological Properties

Regarding left hemispheric gliomas (for detailed results, see **Table 2** and **Figure 1**), the local efficiency (*p* = 0.0138), clustering coefficient (*p* < 0.0001), fault tolerance (*p* = 0.0009), transitivity (*p* < 0.0001), and vulnerability (*p* = 0.0074) were different among the three groups (non-deficit, deficit, and healthy groups). *Post-hoc* analysis with Bonferroni correction showed that the non-deficit group had greater local efficiency than the deficit (*p* = 0.0461) and healthy (*p* = 0.0181) groups. Moreover, compared to the non-deficit group, the clustering coefficient and transitivity decreased in the deficit (*p* < 0.0001 and *p* < 0.0001, respectively) and healthy (*p* = 0.0022 and *p* < 0.0001, respectively) groups. Additionally, compared to the non-deficit group, the fault tolerance increased in the deficit (*p* = 0.0138) and healthy (*p* = 0.0008) groups. Furthermore, the vulnerability was weaker in the deficit (*p* = 0.0077) and healthy (*p* = 0.0439) groups than that in the non-deficit group.

**Table 2:** Global properties compared between the patient and healthy groups for tumors located on the left hemisphere.

	Non-deficit group	Deficit group	Healthy group	One-way ANOVA (*p*-value)	*Post-hoc* analysis (*p*-value)		
					Deficit *vs.* non-deficit	Non-deficit *vs.* healthy	Deficit *vs*. healthy
Local efficiency	0.301 ± 0.007	0.263 ± 0.012	0.264 ± 0.008	0.0138	0.0461	0.0181	>0.9999
Clustering coefficient	0.322 ± 0.014	0.215 ± 0.010	0.256 ± 0.013	<0.0001	<0.0001	0.0022	0.0935
Global efficiency	0.304 ± 0.017	0.285 ± 0.015	0.275 ± 0.009	0.2628	–	–	–
Shortest path length	4.525 ± 0.240	4.748 ± 0.229	4.917 ± 0.153	0.3677	–	–	–
Gamma	1.025 ± 0.020	0.931 ± 0.027	1.000 ± 0.014	0.0289	0.0344	>0.9999	0.0963
Lambda	0.988 ± 0.002	0.988 ± 0.003	0.991 ± 0.002	>0.9999	–	–	–
Sigma	1.039 ± 0.020	0.942 ± 0.037	1.001 ± 0.014	0.0299	0.0271	0.6955	0.1913
Fault tolerance	1.255 ± 0.066	1.475 ± 0.046	1.508 ± 0.035	0.0009	0.0138	0.0008	>0.9999
Transitivity	0.166 ± 0.012	0.094 ± 0.006	0.108 ± 0.007	<0.0001	<0.0001	<0.0001	0.6810
Vulnerability	0.235 ± 0.013	0.178 ± 0.011	0.195 ± 0.010	0.0074	0.0077	0.0439	0.8297

Global properties were calculated with one-way ANOVA. If the results of one-way ANOVA were significant, post-hoc analysis with Bonferroni correction was subsequently applied.

Regarding right hemispheric gliomas (for detailed results, see **Table 3** and **Figure 2**), the local efficiency (*p* = 0.0063), clustering coefficient (*p* = 0.0003), fault tolerance (*p* = 0.0008), transitivity (*p* = 0.0045), and vulnerability (*p* = 0.0130) were different among the three groups. *Post-hoc* analysis with Bonferroni correction revealed that the local efficiency was weaker in the deficit (*p* = 0.0084) and healthy (*p* = 0.0191) groups than that in the non-deficit group. Moreover, compared with the non-deficit group, the clustering coefficient and transitivity decreased in the deficit (*p* = 0.0002 and *p* = 0.0048, respectively) and healthy (*p* = 0.0386 and *p* = 0.0199, respectively) groups. Additionally, compared to the non-deficit group, the fault tolerance increased in the deficit (*p* = 0.0035) and healthy (*p* = 0.0005) groups. Furthermore, the vulnerability was weaker in the deficit (*p* = 0.0141) and healthy (*p* = 0.0469) groups than that in the non-deficit group.

**Table 3:** Global properties compared between the patient and healthy groups for tumors located on the right hemisphere.

	Non-deficit group	Deficit group	Healthy group	One-way ANOVA (*p*-value)	*Post-hoc* analysis (*p-*value)		
					Deficit *vs.* non-deficit	Non-deficit *vs.* healthy	Deficit *vs.* healthy
Local efficiency	0.347 ± 0.023	0.273 ± 0.013	0.288 ± 0.011	0.0063	0.0084	0.0191	>0.9999
Clustering coefficient	0.326 ± 0.018	0.233 ± 0.014	0.277 ± 0.010	0.0003	0.0002	0.0386	0.0536
Global efficiency	0.353 ± 0.026	0.293 ± 0.014	0.301 ± 0.012	0.0595	–	–	–
Shortest path length	4.000 ± 0.246	4.610 ± 0.222	4.607 ± 0.153	0.0896	–	–	–
Gamma	1.112 ± 0.017	1.031 ± 0.031	1.006 ± 0.011	0.0002	0.0212	0.0001	>0.9999
Lambda	1.047 ± 0.011	1.041 ± 0.035	1.000 ± 0.012	0.0582	–	–	–
Sigma	1.062 ± 0.013	0.986 ± 0.013	1.006 ± 0.011	<0.0001	0.0233	0.0498	>0.9999
Fault tolerance	1.232 ± 0.045	1.441 ± 0.070	1.517 ± 0.036	0.0008	0.0035	0.0005	0.7915
Transitivity	0.172 ± 0.017	0.112 ± 0.011	0.127 ± 0.008	0.0045	0.0048	0.0199	0.9784
Vulnerability	0.254 ± 0.017	0.196 ± 0.008	0.211 ± 0.010	0.0130	0.0141	0.0469	>0.9999

Global properties were calculated with one-way ANOVA. If the results of one-way ANOVA were significant, post-hoc analysis with Bonferroni correction was subsequently applied.

### Small-World Properties

There were some differences in the gamma (left-sided glioma, *p* = 0.0289; right-sided glioma, *p* = 0.0001) and sigma (left-sided glioma, *p* = 0.0299; right-sided glioma, *p* = 0.0153) values among the three groups (for detailed results, see **Tables 2** and **3**). However, there was no difference in the lambda (left-sided glioma, *p* > 0.9999; right-sided glioma, *p* > 0.9999) values among the three groups.

Regarding left hemispheric gliomas, the gamma value was greater in the non-deficit group than that in the deficit group (*p* = 0.0344) after *post-hoc* analysis with Bonferroni correction. Moreover, the sigma value was greater in the non-deficit group than that in the deficit group (*p* = 0.0271).

Regarding right hemispheric gliomas, the gamma value was greater in the non-deficit group than that in the deficit (*p* = 0.0212) and healthy (*p* = 0.0001) groups after *post-hoc* analysis with Bonferroni correction. Moreover, the sigma value was greater in the non-deficit group than that in the deficit (*p* = 0.0233) and healthy (*p* = 0.0498) groups.

### Nodal Topological Properties

**Figure 3:**
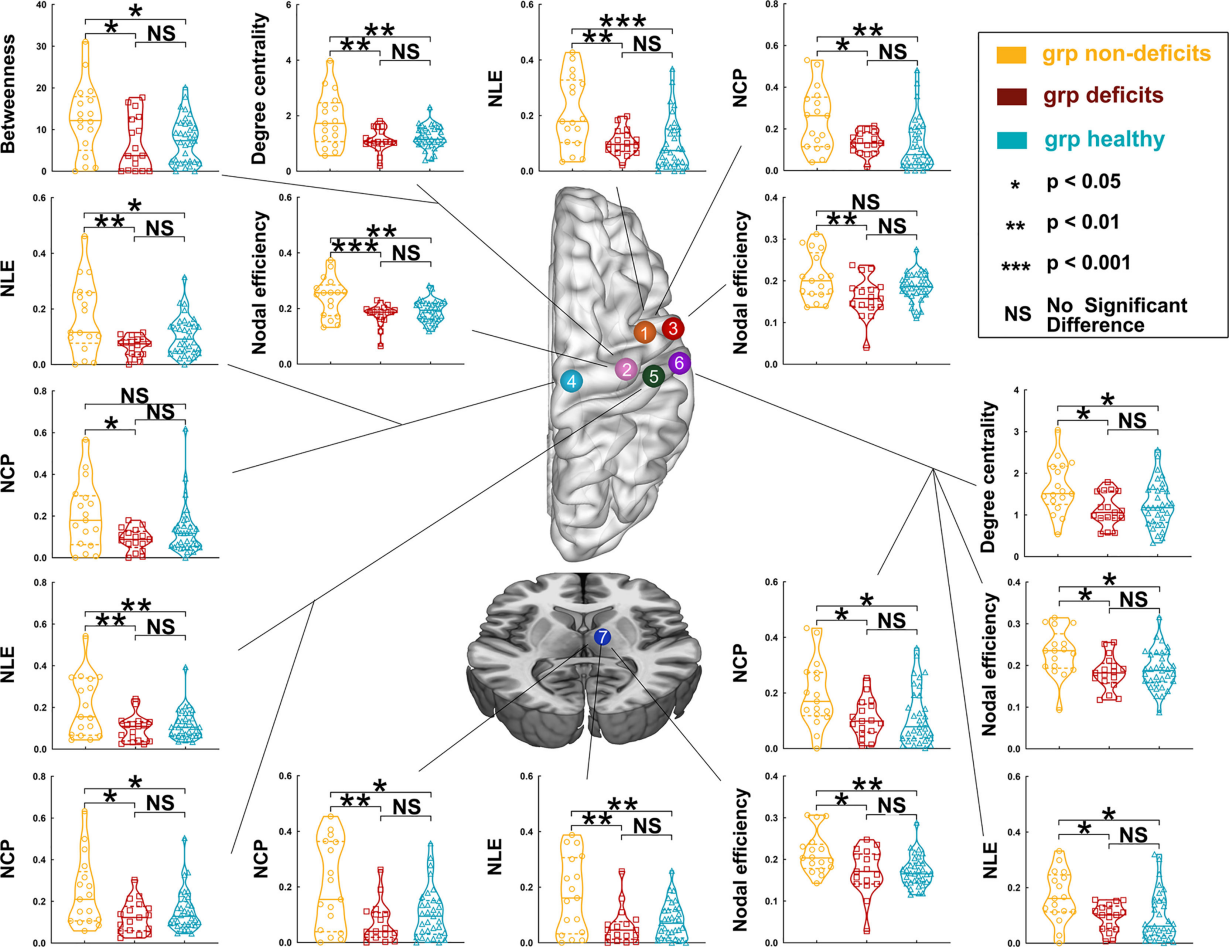
Differences in nodal topological properties of the sensorimotor network in the contralesional hemisphere among the three groups, with left hemisphere glioma. *Orange node* (No. 1), caudal dorsolateral Brodmann area (BA) 6 (A6cdl); *pink node* (No. 2), upper limb of BA 4 (A4ul); *red node* (No. 3), tongue and larynx of BA 4 (A4tl); *light blue node* (No. 4), lower limb region of BA 4 (A4ll); *green node* (No. 5), upper limb, head, and face regions of BA 1/2/3 (A1/2/3ulhf); *purple node* (No. 6), tongue and larynx of BA 1/2/3 (A1_2_3tonIa); and *dark blue node* (No. 7), premotor-related thalamus (mPMtha).

Regarding left hemispheric gliomas, among the three groups (for detailed results, see **Supplementary Tables S3–S7** and **Figure 3**), the nodal local efficiency values of five nodes—caudal dorsolateral Brodmann area (BA) 6 (A6cdl), lower limb BA 4 (A4ll), upper limb and face BA 1/2/3 (A1_2_3ulhf), tongue and larynx of BA 1/2/3 (A1_2_3tonIa), and premotor thalamus (mPMtha)—were significantly altered on *post-hoc* tests with Bonferroni correction. Compared with the non-deficit group, the nodal local efficiency of these nodes decreased in the deficit (A6cdl, *p* = 0.0048; A4ll, *p* = 0.0014; A1_2_3ulhf, *p* = 0.0044; A1_2_3tonIa, *p* = 0.0270; and mPMtha, *p* = 0.0011) and healthy (A6cdl, *p* = 0.0005; A4ll, *p* = 0.0278; A1_2_3ulhf, *p* = 0.0075; A1_2_3tonIa, *p* = 0.0119; and mPMtha, *p* = 0.0019) groups. Similarly, the nodal clustering coefficients of these nodes (except node A4ll) were greater in the non-deficit group than those in the deficit (A6cdl, *p* = 0.0212; A1_2_3ulhf, *p* = 0.0113; A1_2_3tonIa, *p* = 0.0394; and mPMtha, *p* = 0.0027) and healthy (A6cdl, *p* = 0.0034; A1_2_3ulhf, *p* = 0.0342; A1_2_3tonIa, *p* = 0.0300; and mPMtha, *p* = 0.0138) groups. Moreover, the nodal efficiency values of three nodes—upper limb BA 4 (A4ul), A1_2_3tonIa, and mPMtha—were significantly altered. Compared with the non-deficit group, the nodal efficiency of these nodes decreased in the deficit (A4ul, *p* = 0.0007; A1_2_3tonIa, *p* = 0.0134; and mPMtha, *p* = 0.0182) and healthy (A4ul, *p* = 0.0030; A1_2_3tonIa, *p* = 0.0174; and mPMtha, *p* = 0.0058) groups. Additionally, the degree centrality of two nodes (A4ul and A1_2_3tonIa) was significantly altered. Compared with the non-deficit group, the degree centrality decreased in the deficit (A4ul, *p* = 0.0020; A1_2_3tonIa, *p* = 0.0146) and healthy (A4ul, *p* = 0.0023; A1_2_3tonIa, *p* = 0.0436) groups. Furthermore, the betweenness centrality of node A4ul was significantly altered. Compared with the non-deficit group, the betweenness centrality decreased in the deficit (*p* = 0.0379) and healthy (*p* = 0.0402) groups.

**Figure 4:**
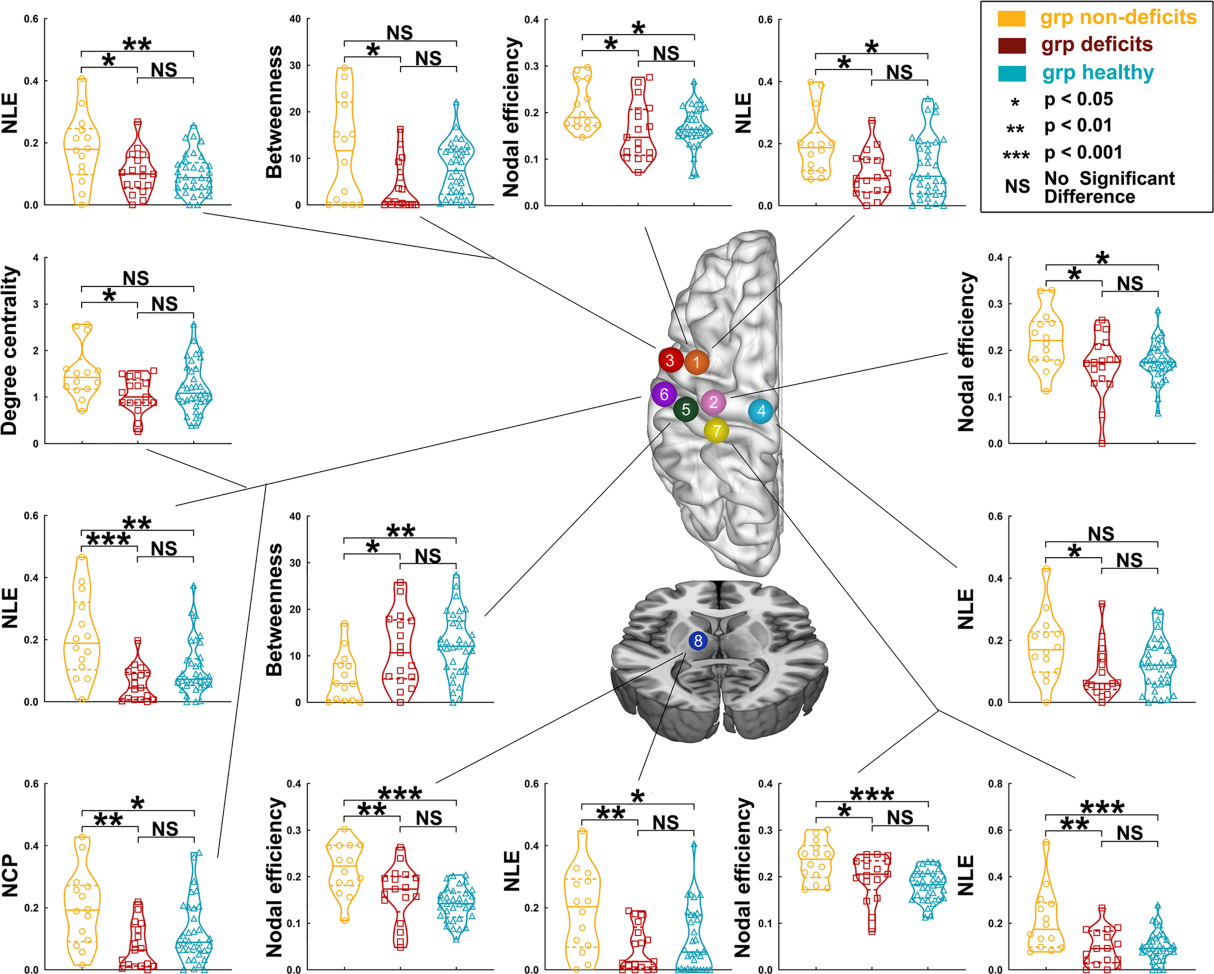
Differences in nodal topological properties of the sensorimotor network in the contralesional hemisphere among the three groups, with right hemisphere glioma. *Orange node* (No. 1), caudal dorsolateral Brodmann area (BA) 6 (A6cdl); *pink node* (No. 2), upper limb of BA 4 (A4ul); *red node* (No. 3), tongue and larynx of BA 4 (A4tl); *light blue node* (No. 4), lower limb region of BA 4 (A4ll); *green node* (No. 5), upper limb, head, and face regions of BA 1/2/3 (A1/2/3ulhf); *purple node* (No. 6), tongue and larynx of BA 1/2/3 (A1_2_3tonIa); *yellow node* (No. 7), trunk region of BA 1/2/3 (A1_2_3tru); and *dark blue node* (No. 8), premotor-related thalamus (mPMtha).

Regarding right hemispheric gliomas, among the three groups (for detailed results, see **Supplementary Tables S8–S12** and **Figure 4**), the nodal local efficiency values of four nodes—A6cdl, A4tl, A1_2_3tonIa, and mPMtha—were significantly altered on *post-hoc* tests with Bonferroni correction. Compared with the non-deficit group, the nodal local efficiency values of these nodes decreased in the deficit (A6cdl, *p* = 0.0172; A4tl, *p* = 0.0210; A1_2_3tonIa, *p* < 0.0001; and mPMtha, *p* = 0.0056) and healthy (A6cdl, *p* = 0.0492; A4tl, *p* = 0.0057; A1_2_3tonIa, *p* = 0.0027; and mPMtha, *p* = 0.0158) groups. The nodal clustering coefficient of node A1_2_3tonIa was greater in the non-deficit group than that in the deficit (*p* = 0.0027) and healthy (*p* = 0.0500) groups. Moreover, the nodal efficiency values of four nodes—A6cdl, A4ul, trunk BA 1/2/3 (A1_2_3tru), and mPMtha—were significantly altered. Compared with the non-deficit group, the nodal efficiency of these nodes decreased in the deficit (A6cdl, *p* = 0.0187; A4ul, *p* = 0.0175; A1_2_3tru, *p* = 0.0445; and mPMtha, *p* = 0.0065) and healthy (A6cdl, *p* = 0.0213; A4ul, *p* = 0.0215; A1_2_3tru, *p* = 0.0003; and mPMtha, *p* < 0.0001) groups. Additionally, the degree centrality of node A1_2_3tonIa was significantly altered. Compared with the non-deficit group, the degree centrality decreased in the deficit group (*p* = 0.0248). Furthermore, the betweenness centrality of node A1_2_3ulhf was significantly altered. Compared with the non-deficit group, the betweenness centrality decreased in the deficit (*p* = 0.0468) and healthy (*p* = 0.0050) groups.

### Correlation Between Muscle Strength and Topological Properties

Regarding patients with left glioma, the clustering coefficient (*r* = 0.805, *p* < 0.0001), local efficiency (*r* = 0.356, *p* = 0.0390), gamma (*r* = 0.695, *p* < 0.0001) and sigma (*r* = 0.677, *p* < 0.0001) values, transitivity (*r* = 0.741, *p* < 0.0001), and vulnerability (*r* = 0.540, *p* = 0.0010) were positively correlated with muscle strength of deficit hand. Similarly, regarding patients with right glioma, the clustering coefficient (*r* = 0.704, *p* < 0.0001), local efficiency (*r* = 0.477, *p* = 0.0067), gamma (*r* = 0.702, *p* < 0.0001) and sigma (*r* = 0.711, *p* < 0.0001) values, transitivity (*r* = 0.603, *p* = 0.0003), and vulnerability (*r* = 0.510, *p* = 0.0034) were positively correlated with the muscle strength of deficit hand (**Supplementary Figure S1**).

## Discussion

This study investigated the alterations of the cSMN in patients with gliomas and with different preoperative motor status. The results revealed that the FC of the cSMN was not different between the patient and healthy groups (for detailed information, see part 2 of the **Supplementary Material**). However, the topological properties of the cSMN were significantly different between patients with and without motor deficits.

In our study, the network properties (such as the clustering coefficient and local efficiency) in the non-deficit group were greater than those in the healthy group. The clustering coefficient represents the ratio between the number of actual edges and the maximum number of possible edges in a network (26). Thus, compared with the healthy group, the increased clustering coefficient in the non-deficit group meant that the network was remodeled through increasing the number of actual edges. The remodeling networks implied that the mirrored motor cortices on the contralesional hemisphere participated in functional compensation in the non-deficit group, and this compensation process resulted in normal motor function when the glioma involved the primary motor area (2, 7, 30).

How do contralesional cortices compensate for motor function? The tendency of alterations of the topological properties among the three groups indicated that two stages of motor functional compensation might exist.

In our study, the cSMN in the non-deficit group showed small-world properties and was significantly increased compared with that of the healthy group, but the cSMN in the deficit group did not have small-world properties (*σ* < 1). Small-world properties were calculated based on the clustering coefficient and shortest path length (31–33). The small-world properties were found to be increased in the non-deficit group and decreased in the deficit group compared with the healthy group. Specifically, the clustering coefficient was changed and the shortest path length was unchanged. The inverse tendency of alterations of the clustering coefficients between the non-deficit and deficit groups was determined by the different degrees of network disruption. The network disruptions were more serious in the deficit group than those in the non-deficit group, as patients in the deficit group had larger tumor volumes (30, 34, 35). The different degrees of network disruptions also explained that the local efficiency, clustering coefficient, gamma and sigma values, and transitivity were positively correlated with the muscle strength of contralesional hemisphere limbs. Moreover, the contralesional primary motor area is necessary for recovery if a lesion invaded large parts of the motor area (36). Since the contralateral cortex is persistently involved in compensating for impaired motor function, we believed that the reason patients suffer from preoperative motor deficits was that this compensated motor function was further damaged by growing gliomas. In addition, the shortest path length was insignificantly changed both in the deficit and non-deficit groups. Accordingly, similar to stroke patients increasing the small-worldness of the network after recovery training (29). Based on the evidence we found in patients with glioma, we hypothesized that the cSMN firstly acquired a high conveying efficiency (improved small-worldness of the cSMN) to participate in compensating for the damaged motor function in the lesional hemisphere (non-deficit stage). Subsequently, the cSMN developed a low conveying efficiency (reduced small-worldness in the cSMN) because the compensatory motor function was further damaged by the growing glioma (deficit stage). Furthermore, this finding does not contradict the conventional theory that motor function is controlled by the contralateral hemisphere. Despite the participation of the contralesional hemisphere in motor functional compensation, patients would still suffer from motor deficits as the disease progresses in the lesional hemisphere.

Vulnerability evaluates the stability of a network (14), which represents the alterations of the conveying efficiency if each node in a network is replaced (28, 37). In our study, the vulnerability of the cSMN increased in the non-deficit group, but was insignificantly altered in the deficit group. This finding indicates that the cSMN in the deficit group was more stable than that in the non-deficit group.

Tumor grade is not a determined factor for the different network alterations in the deficit and non-deficit groups. The theory of compensatory functions in glioma patients is that, when a glioma appears, the functional network begins remodeling to compensate for the damaged functions (8). This compensation firstly recruits tissues surrounding the glioma, and then the homotopic areas in the contralesional hemisphere if the damaged function cannot be compensated by surrounding tissues (3). This theory supports the existence of the compensatory stage of motor function in glioma patients. Some studies revealed that high-grade gliomas (HGGs), as well as low-grade gliomas (LGGs), were able to induce contralateral network reorganization (38–41) and neuroplasticity (42–44). Unlike stoke, gliomas are relatively slow growing; therefore, the brain tissue around the tumor has enough time to develop neuroplasticity, which may occur earlier in HGG than in LGG due to its high invasiveness (2, 40). In order to identify the differences of brain networks between the compensatory and decompensated stages, patients with HGGs are a good model since those with LGGs often have normal function at surgery. Moreover, the clinical manifestations of patients showed that the influence of HGGs is also gradually aggravated (45). To avoid bias, the tumor volume and tumor grade were regressed out as covariates in this study.

Some patients with HGGs suffered from motor deficits, which meant that the compensation of motor function was insufficient (46). Therefore, combined with the alterations of conveying efficiency in the different stages (compensatory and decompensatory stages), we concluded that the cSMN sacrificed stability to exchange conveying efficiency in order to compensate for the damaged motor function in the compensatory stage. Meanwhile, the healthy hemisphere maintained its own motor function with low conveying efficiency. In the decompensatory stage, when the motor function in the lesional hemisphere was completely destroyed by the growing glioma, the conveying efficiency of the contralesional hemisphere was unable to recover and remained low, and the cSMN returned to a stable status.

Regarding the alterations in nodal properties, we found that most of the alterations focused on the primary sensorimotor areas and motor-related thalamus. We found an increasing tendency of alterations of nodal properties in the non-deficit group and an unchanging tendency in the deficit group compared with the healthy group. This finding further verified that the contralesional sensorimotor cortices are crucial for motor functional compensation (47, 48). Moreover, our results showed that, regardless of the glioma location, the nodal efficiency, nodal clustering coefficient, and nodal local efficiency of the nodes (premotor-related thalamus) increased in the non-deficit group and decreased in the deficit group. These findings indicated that the premotor-related thalamus played an important role in compensating for the damaged motor function in the non-deficit stage and significantly decreased the conveying efficiency in the deficit stage. Previous studies showed that the contralesional motor-related thalamus participated in motor plasticity by changing pathways and building midline-crossing contralesional corticospinal fibers (47, 48). Hence, our findings verified this theory through functional networks and differed from previous verification through structural networks. Furthermore, as previous studies have shown, the motor-related thalamus is crucial for the modulation of motor function (30, 49–51). Our findings might provide a target for further protection in operations and for stimulating treatment using repetitive transcranial magnetic stimulation post-operation.

An important point that should be raised is that our study did not investigate the interhemispheric alterations of FCs or topological properties in order to avoid negative effects from neurovascular uncoupling and tumor occupation as much as possible. In this study, we chose the cSMN because the origin of blood supply in each hemisphere is different. Consequently, the interhemispheric FC, as well as the topological properties of the entire sensorimotor network, could not be investigated. Previous studies showed that neurovascular uncoupling affects the results of rs-fMRI (52), but we considered that their conclusions were biased due to the calculation of interhemispheric FCs (53). In fact, no significant difference in the FCs of the cSMN between the patient and healthy groups was found in previous glioma-relevant studies (53–55). Our findings in FCs are consistent with these previous studies.

The most important limitation of this study is that, although our findings are encouraging, they are still conjectures based on the controversial theory that the symmetric cortex is able to compensate for the damaged function in the lesional hemisphere (55, 56). The use of transcranial magnetic stimulation may validate our findings in the future.

In summary, alteration of the topological properties of the cSMN is a dynamic process in patients with gliomas. Two stages of motor functional alterations may exist. In the compensatory stage, the cSMN sacrificed stability to acquire high conveying efficiency and to compensate for the damaged motor function of the lesional hemisphere. With tumor growth and deterioration, patients enter the decompensatory stage, and motor dysfunction on the affected side arises. The cSMN returns to a stable state and maintains healthy hemispheric motor function, but with low efficiency.

## Data Availability Statement

The raw data supporting the conclusions of this article will be made available by the authors, without undue reservation.

## Ethics Statement

The studies involving human participants were reviewed and approved by the IRB of Beijing Tiantan Hospital. The patients/participants provided written informed consent to participate in this study.

## Author Contributions

SF, LL, SW, and YG: study concept and design. SF, LL, SW, ZZ, and YG: data acquisition and analysis. SF, XF, and YW: statistics/verified analytical method. SF, XF, and YW: writing the first draft. XF, YW, and TJ: study supervision. All authors contributed to the article and approved the submitted version.

## Funding

This work was supported by the Public Welfare Development and Reform Pilot Project of Beijing Medical Research Institute (PXM2019_026280_000008), Beijing Municipal Natural Science Foundation (No. 7202021), National Natural Science Foundation of China (No. 82001777), and the Research Unit of Accurate Diagnosis, Treatment, and Translational Medicine of Brain Tumors (No. 2019-I2M-5-021).

## Conflict of Interest

The authors declare that the research was conducted in the absence of any commercial or financial relationships that could be construed as a potential conflict of interest.

## Publisher’s Note

All claims expressed in this article are solely those of the authors and do not necessarily represent those of their affiliated organizations, or those of the publisher, the editors and the reviewers. Any product that may be evaluated in this article, or claim that may be made by its manufacturer, is not guaranteed or endorsed by the publisher.
