# Supplementary material for: Contralesional Sensorimotor Network Participates in Motor Functional Compensation in Glioma Patients
Source: Front Oncol. 2022 Apr 22;12:882313. doi: 10.3389/fonc.2022.882313 (PMC9072743; doi:10.3389/fonc.2022.882313)
Supplement: Supplementary file 1 [file DataSheet_1.pdf]

Title: Contralesional sensorimotor network participates in motor functional compensation in glioma patients

Supplementary Materials

Part 1. Differences in functional connectivity

Compared with the healthy group, no difference in functional connectivity (FC) was found between the deficit and non-deficit groups after Bonferroni correction (*p*-value threshold = 5.49×10^{-4}), regardless of whether the glioma was located in the left or right hemisphere. When gliomas grew in the left hemisphere, only one edge had an increased FC in the non-deficit group (0.235 ± 0.049) compared with that in the deficit group (-0.007 ± 0.040) after Bonferroni correction. The altered edge was connected to the node of A4ul_R (Brodmann area [BA] 4 [upper limb]) to the node of A4ll_R (BA 4 [lower limb], $p = 0.0002$).

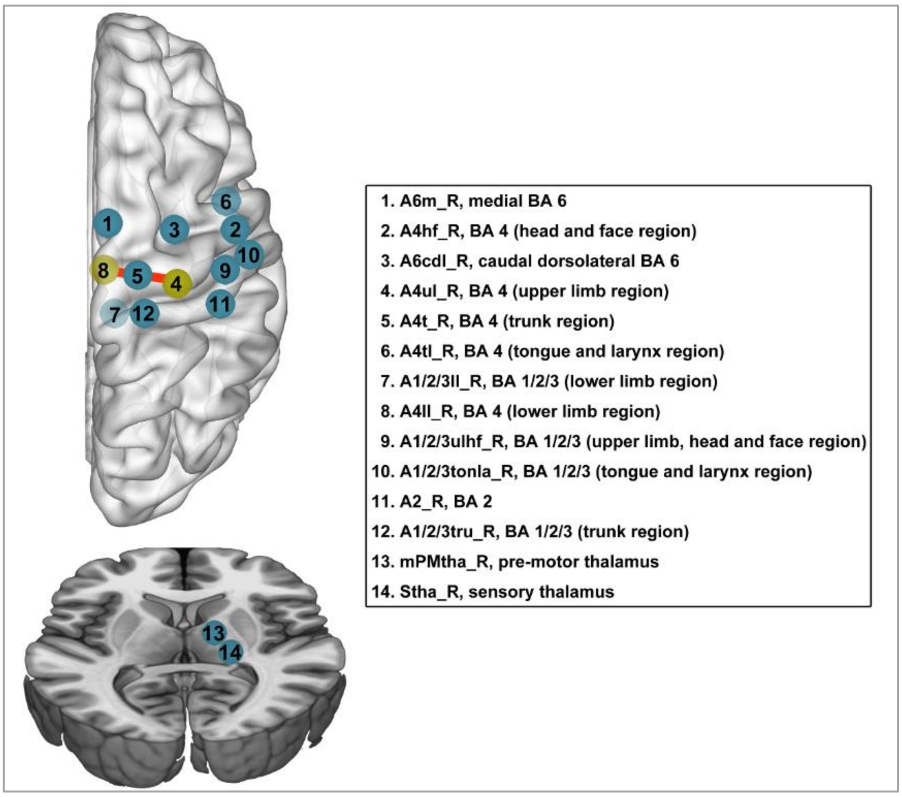

Figure. Alterations of functional connectivity in the contralesional hemisphere with left hemisphere glioma. The red line represents that functional connectivity significantly increased in the non-deficit group compared with the deficit group.

Part 2. Supplemental Tables

Table S1. Montreal Neurological Institute (MNI) locations of 14 nodes in the right hemispheric sensorimotor network

Regions of interesting	Modified Cyto-architectonic	Right hemisphere		
		X	Y	Z
A6m_R	Medial area BA 6	6	38	35
A4hf_R	Area BA 4 (head and face)	55	-2	33
A4ul_R	Area BA 4 (upper limb)	34	-19	59
A4t_R	Area BA 4 (trunk)	15	-22	71
A4tl_R	Area BA 4 (tongue and larynx)	54	4	9
A6cvl_R	caudal ventrolateral Area BA 6	51	7	30
A1_2_3ll_R	Area BA 1/2/3 (lower limb)	10	-34	54
A4ll_R	Area BA 4 (lower limb)	5	-21	61
A1_2_3ulhf_R	Area BA 1/2/3 (upper limb and face)	50	-14	44
A1_2_3tonIa_R	Area BA 1/2/3 (tongue and larynx)	56	-10	15
A2_R	Area BA 2	48	-24	48
A1_2_3tru_R	Area BA 1/2/3 (trunk)	20	-33	69
mPMtha_R	Pre-motor thalamus	12	-14	1
Stha_R	Sensory thalamus	18	-22	3

*****BA = Brodmann area.

Table S2. Montreal Neurological Institute (MNI) locations of 14 nodes in the left hemispheric sensorimotor network

Regions of interesting	Modified Cyto-architectonic	Left hemisphere		
		X	Y	Z
A6m_L	Medial area BA 6	5	36	38
A4hf_L	Area BA 4 (head and face)	-49	-8	39
A4ul_L	Area BA 4 (upper limb)	-26	-25	63
A4t_L	Area BA 4 (trunk)	-13	-20	73
A4tl_L	Area BA 4 (tongue and larynx)	-52	0	8
A6cvl_L	caudal ventrolateral Area BA 6	-49	5	30
A1_2_3ll_L	Area BA 1/2/3 (lower limb)	-8	-38	58
A4ll_L	Area BA 4 (lower limb)	-4	-23	61
A1_2_3ulhf_L	Area BA 1/2/3 (upper limb and face)	-50	-16	43
A1_2_3tonIa_L	Area BA 1/2/3 (tongue and larynx)	-56	-14	16
A2_L	Area BA 2	-46	-30	50
A1_2_3tru_L	Area BA 1/2/3 (trunk)	-21	-35	68
mPMtha_L	Pre-motor thalamus	-18	-23	3
Stha_L	Sensory thalamus	-18	-14	4

*****BA = Brodmann area.

Table S3. Nodal local efficiency compared between the patients and healthy groups for tumor locating on the left hemisphere

Node name	non-Deficits group	Deficits group	Health group	One-way ANOVA (p value)	Post-hoc analysis (p value)		
					Deficits vs non-Deficits	non-Deficits vs Health	Deficits vs Health
A6m_R	0.127 ± 0.026	0.077 ± 0.018	0.118 ± 0.014	0.2189	-	-	-
A4hf_R	0.133 ± 0.026	0.115 ± 0.018	0.101 ± 0.015	0.5132	-	-	-
A6cdl_R	0.213 ± 0.030	0.104 ± 0.011	0.098 ± 0.016	0.0004	0.0048	0.0005	> 0.9999
A4ul_R	0.147 ± 0.023	0.090 ± 0.015	0.116 ± 0.015	0.1514	-	-	-
A4t_R	0.130 ± 0.030	0.090 ± 0.015	0.114 ± 0.012	0.4109	-	-	-
A4tl_R	0.156 ± 0.027	0.119 ± 0.023	0.108 ± 0.015	0.2559	-	-	-
A1_2_3ll_R	0.128 ± 0.027	0.088 ± 0.020	0.088 ± 0.012	0.2706	-	-	-
A4ll_R	0.171 ± 0.031	0.064 ± 0.008	0.103 ± 0.012	0.0018	0.0014	0.0278	0.3821
A1_2_3ulhf_R	0.207 ± 0.034	0.099 ± 0.015	0.118 ± 0.012	0.0024	0.0044	0.0075	> 0.9999
A1_2_3tonIa_R	0.168 ± 0.022	0.093 ± 0.011	0.096 ± 0.015	0.0082	0.0270	0.0119	> 0.9999
A2_R	0.167 ± 0.029	0.104 ± 0.020	0.112 ± 0.020	0.1819	-	-	-
A1_2_3tru_R	0.172 ± 0.029	0.0945 ± 0.017	0.138 ± 0.015	0.0634	-	-	-
mPMtha_R	0.173 ± 0.032	0.059 ± 0.016	0.078 ± 0.011	0.0005	0.0011	0.0019	> 0.9999
Stha_R	0.153 ± 0.029	0.116 ± 0.023	0.070 ± 0.012	0.0132	0.7683	0.0126	0.3106

*****The global properties were calculated with one-way ANOVA test. If the results one-way ANOVA were significance, post-hoc analysis with bonferroni correction was subsequently applied.

Table S4. Nodal cluster coefficient compared between the patients and healthy groups for tumor locating on the left hemisphere

Node name	non-Deficits group	Deficits group	Health group	One-way ANOVA (p value)	Post-hoc analysis (p value)		
					Deficits vs non-Deficits	non-Deficits vs Health	Deficits vs Health
A6m_R	0.160 ± 0.038	0.092 ± 0.018	0.141 ± 0.019	0.2263	-	-	-
A4hf_R	0.165 ± 0.034	0.143 ± 0.023	0.123 ± 0.016	0.4375	-	-	-
A6cdl_R	0.248 ± 0.035	0.135 ± 0.012	0.128 ± 0.021	0.0031	0.0212	0.0034	> 0.9999
A4ul_R	0.157 ± 0.025	0.101 ± 0.017	0.141 ± 0.016	0.1816	-	-	-
A4t_R	0.161 ± 0.035	0.112 ± 0.018	0.152 ± 0.017	0.3577	-	-	-
A4tl_R	0.193 ± 0.031	0.131 ± 0.022	0.145 ± 0.021	0.2612	-	-	-
A1_2_3ll_R	0.157 ± 0.036	0.113 ± 0.026	0.112 ± 0.014	0.3534	-	-	-
A4ll_R	0.204 ± 0.038	0.088 ± 0.012	0.130 ± 0.020	0.0205	0.0189	0.1312	0.7172
A1_2_3ulhf_R	0.243 ± 0.039	0.125 ± 0.018	0.154 ± 0.017	0.0088	0.0113	0.0342	> 0.9999
A1_2_3tonIa_R	0.195 ± 0.028	0.108 ± 0.016	0.116 ± 0.017	0.0175	0.0394	0.0300	> 0.9999
A2_R	0.194 ± 0.034	0.111 ± 0.020	0.141 ± 0.019	0.1099	-	-	-
A1_2_3tru_R	0.201 ± 0.034	0.124 ± 0.023	0.170 ± 0.019	0.1604	-	-	-
mPMtha_R	0.200 ± 0.037	0.071 ± 0.017	0.105 ± 0.016	0.0022	0.0027	0.0138	0.8726
Stha_R	0.183 ± 0.036	0.162 ± 0.032	0.086 ± 0.013	0.0117	> 0.9999	0.0206	0.0958

*****The global properties were calculated with one-way ANOVA test. If the results one-way ANOVA were significance, post-hoc analysis with bonferroni correction was subsequently applied.

Table S5. Nodal efficiency compared between the patients and healthy groups for tumor locating on the left hemisphere

Node name	non-Deficits group	Deficits group	Health group	One-way ANOVA (p value)	Post-hoc analysis (p value)		
					Deficits vs non-Deficits	non-Deficits vs Health	Deficits vs Health
A6m_R	0.190 ± 0.019	0.177 ± 0.008	0.186 ± 0.007	0.7662	-	-	-
A4hf_R	0.199 ± 0.017	0.199 ± 0.008	0.177 ± 0.008	0.2470	-	-	-
A6cdl_R	0.214 ± 0.015	0.176 ± 0.011	0.176 ± 0.009	0.0389	0.1080	0.0502	> 0.9999
A4ul_R	0.241 ± 0.017	0.177 ± 0.009	0.191 ± 0.007	< 0.0001	0.0007	0.0030	0.9413
A4t_R	0.218 ± 0.011	0.198 ± 0.010	0.191 ± 0.007	0.1160	-	-	-
A4tl_R	0.209 ± 0.013	0.160 ± 0.011	0.185 ± 0.006	0.0072	0.0053	0.2361	0.1610
A1_2_3ll_R	0.205 ± 0.014	0.167 ± 0.012	0.176 ± 0.006	0.0590	-	-	-
A4ll_R	0.225 ± 0.011	0.192 ± 0.014	0.198 ± 0.007	0.0796	-	-	-
A1_2_3ulhf_R	0.226 ± 0.012	0.196 ± 0.008	0.207 ± 0.005	0.0547	-	-	-
A1_2_3tonIa_R	0.231 ± 0.013	0.182 ± 0.010	0.190 ± 0.008	0.0071	0.0134	0.0174	> 0.9999
A2_R	0.208 ± 0.010	0.190 ± 0.007	0.187 ± 0.007	0.2063	-	-	-
A1_2_3tru_R	0.229 ± 0.016	0.208 ± 0.008	0.194 ± 0.007	0.0476	0.1880	0.0141	0.3238
mPMtha_R	0.215 ± 0.012	0.171 ± 0.013	0.171 ± 0.006	0.0200	0.0182	0.0058	> 0.9999
Stha_R	0.184 ± 0.012	0.192 ± 0.010	0.160 ± 0.006	0.0255	0.5795	0.0598	0.0130

*****The global properties were calculated with one-way ANOVA test. If the results one-way ANOVA were significance, post-hoc analysis with bonferroni correction was subsequently applied.

Table S6. Degree centrality compared between the patients and healthy groups for tumor locating on the left hemisphere

Node name	non-Deficits group	Deficits group	Health group	One-way ANOVA (p value)	Post-hoc analysis (p value)		
					Deficits vs non-Deficits	non-Deficits vs Health	Deficits vs Health
A6m_R	1.195 ± 0.195	1.073 ± 0.085	1.240 ± 0.079	0.6046	-	-	-
A4hf_R	1.220 ± 0.193	1.354 ± 0.096	1.054 ± 0.076	0.1869	-	-	-
A6cdl_R	1.404 ± 0.189	1.045 ± 0.112	1.063 ± 0.092	0.1281	-	-	-
A4ul_R	1.855 ± 0.225	1.123 ± 0.097	1.227 ± 0.070	0.0008	0.0020	0.0023	> 0.9999
A4t_R	1.368 ± 0.132	1.319 ± 0.122	1.238 ± 0.091	0.6981	-	-	-
A4tl_R	1.326 ± 0.166	0.920 ± 0.110	1.159 ± 0.063	0.0625	-	-	-
A1_2_3ll_R	1.183 ± 0.169	0.951 ± 0.102	1.042 ± 0.068	0.4016	-	-	-
A4ll_R	1.470 ± 0.113	1.267 ± 0.122	1.417 ± 0.085	0.4583	-	-	-
A1_2_3ulhf_R	1.565 ± 0.124	1.300 ± 0.082	1.434 ± 0.067	0.1917	-	-	-
A1_2_3tonIa_R	1.656 ± 0.148	1.122 ± 0.091	1.256 ± 0.094	0.0117	0.0146	0.0436	>0.9999
A2_R	1.325 ± 0.138	1.234 ± 0.098	1.147 ± 0.086	0.4937	-	-	-
A1_2_3tru_R	1.603 ± 0.189	1.429 ± 0.095	1.307 ± 0.075	0.1969	-	-	-
mPMtha_R	1.360 ± 0.151	1.121 ± 0.126	1.037 ± 0.067	0.1047	-	-	-
Stha_R	1.013 ± 0.118	1.259 ± 0.120	0.840 ± 0.063	0.0093	0.3307	0.5908	0.0071

*****The global properties were calculated with one-way ANOVA test. If the results one-way ANOVA were significance, post-hoc analysis with bonferroni correction was subsequently applied.

Table S7. Betweenness centrality compared between the patients and healthy groups for tumor locating on the left hemisphere

Node name	non-Deficits group	Deficits group	Health group	One-way ANOVA (p value)	Post-hoc analysis (p value)		
					Deficits vs non-Deficits	non-Deficits vs Health	Deficits vs Health
A6m_R	7.733 ± 2.638	5.984 ± 1.388	7.856 ± 1.042	0.6959	-	-	-
A4hf_R	5.029 ± 1.748	6.738 ± 1.206	4.809 ± 0.816	0.5020	-	-	-
A6cdl_R	5.125 ± 1.561	5.792 ± 1.269	6.024 ± 1.193	0.8982	-	-	-
A4ul_R	12.471 ± 2.029	6.623 ± 1.517	7.424 ± 0.944	0.0203	0.0379	0.0402	> 0.9999
A4t_R	7.395 ± 1.400	8.755 ± 1.614	7.235 ± 1.145	0.7266	-	-	-
A4tl_R	4.909 ± 1.073	4.044 ± 1.193	6.806 ± 1.027	0.2015	-	-	-
A1_2_3ll_R	7.721 ± 1.925	4.623 ± 1.244	5.571 ± 0.929	0.3259	-	-	-
A4ll_R	7.292 ± 1.568	8.348 ± 1.284	10.973 ± 1.570	0.2409	-	-	-
A1_2_3ulhf_R	7.483 ± 1.473	6.037 ± 0.948	10.629 ± 1.168	0.0324	> 0.9999	0.2649	0.0420
A1_2_3tonIa_R	9.966 ± 2.321	5.547 ± 0.881	7.722 ± 1.155	0.2015	-	-	-
A2_R	4.770 ± 1.183	6.243 ± 1.268	7.687 ± 1.319	0.3275	-	-	-
A1_2_3tru_R	9.522 ± 2.043	10.115 ± 1.027	7.104 ± 0.981	0.2137	-	-	-
mPMtha_R	4.667 ± 1.501	7.311 ± 1.490	6.707 ± 1.011	0.4096	-	-	-
Stha_R	3.958 ± 1.177	7.522 ± 1.323	3.966 ± 0.876	0.0594	-	-	-

*****The global properties were calculated with one-way ANOVA test. If the results one-way ANOVA were significance, post-hoc analysis with bonferroni correction was subsequently applied.

Table S8. Nodal local efficiency compared between the patients and healthy groups for tumor locating on the right hemisphere

Node name	non-Deficits group	Deficits group	Health group	One-way ANOVA (p value)	Post-hoc analysis (p value)		
					Deficits vs non-Deficits	non-Deficits vs Health	Deficits vs Health
A6m_L	0.169 ± 0.030	0.116 ± 0.023	0.115 ± 0.014	0.1801	-	-	-
A4hf_L	0.161 ± 0.031	0.136 ± 0.020	0.168 ± 0.021	0.6527	-	-	-
A6cdl_L	0.199 ± 0.027	0.101 ± 0.017	0.124 ± 0.017	0.0152	0.0172	0.0492	> 0.9999
A4ul_L	0.168 ± 0.043	0.125 ± 0.022	0.098 ± 0.010	0.1086	-	-	-
A4t_ L	0.188 ± 0.022	0.133 ± 0.021	0.106 ± 0.015	0.0180	0.2550	0.0143	0.9459
A4tl_ L	0.180 ± 0.029	0.102 ± 0.016	0.100 ± 0.011	0.0051	0.0210	0.0057	> 0.9999
A1_2_3ll_L	0.125 ± 0.028	0.096 ± 0.024	0.106 ± 0.016	0.7151	-	-	-
A4ll_L	0.180 ± 0.028	0.091 ± 0.019	0.121 ± 0.014	0.0236	0.0212	0.1248	0.7674
A1_2_3ulhf_L	0.146 ± 0.031	0.088 ± 0.016	0.119 ± 0.013	0.1760	-	-	-
A1_2_3tonIa_L	0.209 ± 0.034	0.058 ± 0.013	0.106 ± 0.015	0.0001	< 0.0001	0.0027	0.2507
A2_L	0.131 ± 0.023	0.135 ± 0.026	0.173 ± 0.022	0.3949	-	-	-
A1_2_3tru_L	0.211 ± 0.034	0.096 ± 0.018	0.099 ± 0.011	0.0003	0.0014	0.0004	> 0.9999
mPMtha_L	0.170 ± 0.029	0.074 ± 0.017	0.095 ± 0.018	0.0045	0.0056	0.0158	> 0.9999
Stha_L	0.107 ± 0.024	0.092 ± 0.024	0.086 ± 0.014	0.7679	-	-	-

*The global properties were calculated with one-way ANOVA test. If the results one-way ANOVA were significance, post-hoc analysis with bonferroni correction was subsequently applied.

Table S9. Nodal cluster coefficient compared between the patients and healthy groups for tumor locating on the right hemisphere

Node name	non-Deficits group	Deficits group	Health group	One-way ANOVA (p value)	Post-hoc analysis (p value)		
					Deficits vs non-Deficits	non-Deficits vs Health	Deficits vs Health
A6m_L	0.142 ± 0.025	0.117 ± 0.021	0.130 ± 0.019	0.7862	-	-	-
A4hf_L	0.148 ± 0.026	0.166 ± 0.023	0.224 ± 0.033	0.2276	-	-	-
A6cdl_L	0.180 ± 0.021	0.119 ± 0.019	0.123 ± 0.016	0.1040	-	-	-
A4ul_L	0.144 ± 0.029	0.147 ± 0.026	0.116 ± 0.014	0.4797	-	-	-
A4t_ L	0.211 ± 0.033	0.144 ± 0.029	0.123 ± 0.019	0.0799	-	-	-
A4tl_ L	0.156 ± 0.026	0.110 ± 0.021	0.120 ± 0.015	0.3550	-	-	-
A1_2_3ll_L	0.122 ± 0.025	0.112 ± 0.028	0.113 ± 0.015	0.9535	-	-	-
A4ll_L	0.170 ± 0.025	0.108 ± 0.025	0.151 ± 0.017	0.2119	-	-	-
A1_2_3ulhf_L	0.143 ± 0.034	0.107 ± 0.019	0.157 ± 0.018	0.2919	-	-	-
A1_2_3tonIa_L	0.200 ± 0.031	0.075 ± 0.017	0.122 ± 0.017	0.0037	0.0027	0.0500	0.3558
A2_L	0.135 ± 0.029	0.140 ± 0.025	0.197 ± 0.031	0.2939	-	-	-
A1_2_3tru_L	0.187 ± 0.026	0.115 ± 0.022	0.118 ± 0.015	0.0521	-	-	-
mPMtha_L	0.140 ± 0.033	0.124 ± 0.028	0.104 ± 0.020	0.6097	-	-	-
Stha_L	0.128 ± 0.030	0.098 ± 0.020	0.096 ± 0.015	0.5553	-	-	-

* The global properties were calculated with one-way ANOVA test. If the results one-way ANOVA were significance, post-hoc analysis with least significant difference was subsequently applied.

Table S10. Nodal efficiency compared between the patients and healthy groups for tumor locating on the right hemisphere

Node name	non-Deficits group	Deficits group	Health group	One-way ANOVA (p value)	Post-hoc analysis (p value)		
					Deficits vs non-Deficits	non-Deficits vs Health	Deficits vs Health
A6m_L	0.208 ± 0.014	0.189 ± 0.011	0.168 ± 0.008	0.0359	0.1756	0.0042	0.1325
A4hf_L	0.205 ± 0.011	0.216 ± 0.011	0.204 ± 0.006	0.5872	-	-	-
A6cdl_L	0.212 ± 0.013	0.161 ± 0.015	0.168 ± 0.007	0.0109	0.0187	0.0213	> 0.9999
A4ul_L	0.223 ± 0.016	0.168 ± 0.016	0.176 ± 0.007	0.0105	0.0175	0.0215	> 0.9999
A4t_ L	0.226 ± 0.010	0.189 ± 0.011	0.187 ± 0.008	0.0196	0.0654	0.0213	> 0.9999
A4tl_ L	0.223 ± 0.021	0.176 ± 0.013	0.189 ± 0.007	0.0587	-	-	-
A1_2_3ll_L	0.212 ± 0.020	0.192 ± 0.012	0.191 ± 0.008	0.4453	-	-	-
A4ll_L	0.221 ± 0.013	0.186 ± 0.015	0.190 ± 0.010	0.1762	-	-	-
A1_2_3ulhf_L	0.216 ± 0.010	0.214 ± 0.010	0.221 ± 0.007	0.8349	-	-	-
A1_2_3tonIa_L	0.211 ± 0.015	0.194 ± 0.008	0.188 ± 0.007	0.2655	-	-	-
A2_L	0.199 ± 0.015	0.183 ± 0.011	0.185 ± 0.007	0.5756	-	-	-
A1_2_3tru_L	0.234 ± 0.011	0.198 ± 0.011	0.181 ± 0.006	0.0005	0.0445	0.0003	0.4799
mPMtha_L	0.218 ± 0.014	0.163 ± 0.014	0.141 ± 0.006	0.0019	0.0065	< 0.0001	0.4130
Stha_L	0.195 ± 0.019	0.184 ± 0.012	0.146 ± 0.007	0.0069	> 0.9999	0.0161	0.0577

*****The global properties were calculated with one-way ANOVA test. If the results one-way ANOVA were significance, post-hoc analysis with bonferroni correction was subsequently applied.

Table S11. Degree centrality compared between the patients and healthy groups for tumor locating on the right hemisphere

Node name	non-Deficits group	Deficits group	Health group	One-way ANOVA (p value)	Post-hoc analysis (p value)		
					Deficits vs non-Deficits	non-Deficits vs Health	Deficits vs Health
A6m_L	1.496 ± 0.163	1.260 ± 0.132	1.060 ± 0.081	0.0437	0.6104	0.0409	0.7511
A4hf_L	1.364 ± 0.161	1.589 ± 0.125	1.404 ± 0.076	0.3427	-	-	-
A6cdl_L	1.416 ± 0.169	1.135 ± 0.166	1.022 ± 0.084	0.1125	-	-	-
A4ul_L	1.418 ± 0.171	1.187 ± 0.145	1.215 ± 0.082	0.4082	-	-	-
A4t_ L	1.555 ± 0.159	1.287 ± 0.091	1.289 ± 0.107	0.3537	-	-	-
A4tl_ L	1.724 ± 0.261	1.126 ± 0.170	1.341 ± 0.084	0.0585	-	-	-
A1_2_3ll_L	1.420 ± 0.223	1.309 ± 0.135	1.351 ± 0.086	0.8656	-	-	-
A4ll_L	1.627 ± 0.186	1.209 ± 0.193	1.365 ± 0.120	0.3123	-	-	-
A1_2_3ulhf_L	1.347 ± 0.115	1.579 ± 0.137	1.769 ± 0.100	0.0506	-	-	-
A1_2_3tonIa_L	1.544 ± 0.150	1.041 ± 0.088	1.276 ± 0.095	0.0283	0.0248	0.1659	0.6893
A2_L	1.208 ± 0.134	1.235 ± 0.133	1.255 ± 0.081	0.9800	-	-	-
A1_2_3tru_L	1.710 ± 0.146	1.358 ± 0.127	1.219 ± 0.071	0.0057	0.1090	0.0041	0.9713
mPMtha_L	1.261 ± 0.168	1.221 ± 0.170	0.759 ± 0.059	0.0022	> 0.9999	0.0110	0.0138
Stha_L	1.265 ± 0.165	1.119 ± 0.133	0.878 ± 0.083	0.0391	> 0.9999	0.0628	0.2289

*****The global properties were calculated with one-way ANOVA test. If the results one-way ANOVA were significance, post-hoc analysis with bonferroni correction was subsequently applied.

Table S12. Betweenness centrality compared between the patients and healthy groups for tumor locating on the right hemisphere

Node name	non-Deficits group	Deficits group	Health group	One-way ANOVA (p value)	Post-hoc analysis (p value)		
					Deficits vs non-Deficits	non-Deficits vs Health	Deficits vs Health
A6m_L	1.429 ± 0.170	1.189 ± 0.124	1.009 ± 0.076	0.1213	-	-	-
A4hf_L	1.295 ± 0.145	1.532 ± 0.118	1.364 ± 0.072	0.4276	-	-	-
A6cdl_L	1.359 ± 0.154	1.046 ± 0.167	0.980 ± 0.078	0.4837	-	-	-
A4ul_L	1.356 ± 0.167	1.132 ± 0.140	1.136 ± 0.079	0.9544	-	-	-
A4t_ L	1.472 ± 0.138	1.223 ± 0.099	1.243 ± 0.101	0.7979	-	-	-
A4tl_ L	1.641 ± 0.252	1.054 ± 0.154	1.288 ± 0.086	0.0143	0.0114	0.2893	0.2144
A1_2_3ll_L	1.375 ± 0.216	1.261 ± 0.129	1.295 ± 0.088	0.2050	-	-	-
A4ll_L	1.548 ± 0.172	1.162 ± 0.176	1.316 ± 0.117	0.8476	-	-	-
A1_2_3ulhf_L	1.307 ± 0.114	1.525 ± 0.127	1.720 ± 0.094	0.0060	0.0468	0.0050	> 0.9999
A1_2_3tonIa_L	1.335 ± 0.158	1.213 ± 0.111	1.226 ± 0.092	0.1045	-	-	-
A2_L	1.159 ± 0.130	1.169 ± 0.125	1.188 ± 0.081	0.9798	-	-	-
A1_2_3tru_L	1.668 ± 0.137	1.310 ± 0.120	1.172 ± 0.069	0.5442	-	-	-
mPMtha_L	1.211 ± 0.153	1.168 ± 0.162	0.724 ± 0.056	0.0714	-	-	-
Stha_L	1.198 ± 0.155	1.087 ± 0.125	0.811 ± 0.076	0.3071	-	-	-

*****The global properties were calculated with one-way ANOVA test. If the results one-way ANOVA were significance, post-hoc analysis with bonferroni correction was subsequently applied.

Part 3. Supplementary figure

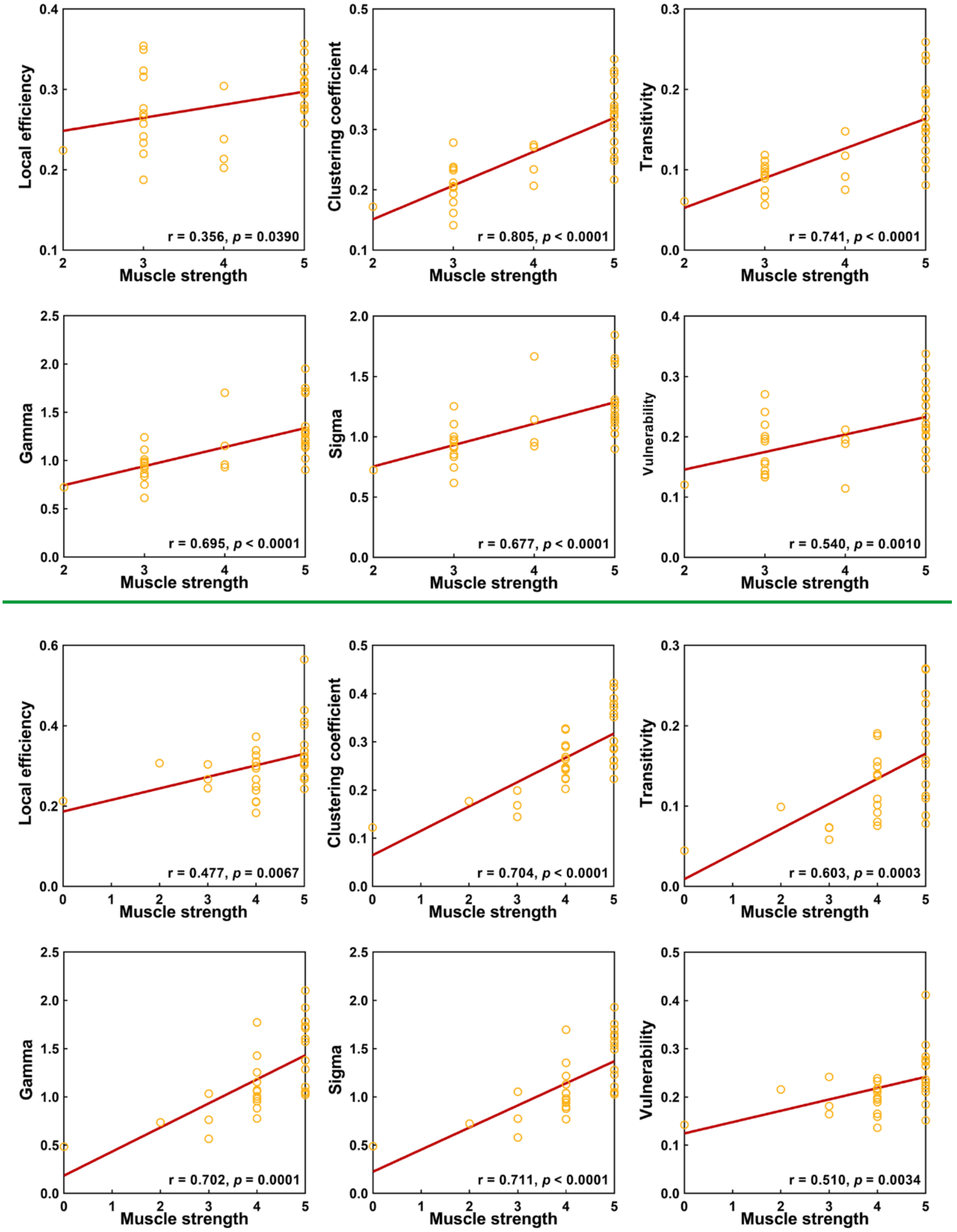

Figure S1. Correlation analysis between topological properties and muscle strength. The upper plane showed the results of patients with left glioma and the under plane showed the results of patients with right glioma.

Reference

1. Fang S, Zhou C, Wang L, Fan X, Wang Y, Zhang Z, et al: Characteristic Alterations of Network in Patients With Intraoperative Stimulation-Induced Seizures During Awake Craniotomy. **Front Neurol 12:**602716, 2021
2. Gong Y, Wu H, Li J, Wang N, Liu H, Tang X: Multi-Granularity Whole-Brain Segmentation Based Functional Network Analysis Using Resting-State fMRI. **Front Neurosci 12:**942, 2018
3. Humphries MD, Gurney K, Prescott TJ: The brainstem reticular formation is a small-world, not scale-free, network. **Proc Biol Sci 273:**503-511, 2006
4. Ji GJ, Yu Y, Miao HH, Wang ZJ, Tang YL, Liao W: Decreased Network Efficiency in Benign Epilepsy with Centrotemporal Spikes. **Radiology 283:**186-194, 2017
